# Feasibility of simultaneous donor nephrectomy and kidney transplantation using a shared single-port robotic platform

**DOI:** 10.1007/s11701-026-03643-8

**Published:** 2026-07-23

**Authors:** Hafiz Umair Siddiqui, Adam Esa, Dylan Isaacson, Sami Shoucair, Venkatesh Krishnamurthi, Jihad Kaouk, Yi-Chia Lin, Alvin Wee, Mohamed Eltemamy

**Affiliations:** 1 Department of Urology, Glickman Urological and Kidney Institute, Cleveland Clinic, 9500 Euclid Avenue, NA-23, Cleveland, OH 44195-3733 USA; 2 Urology Department of Kasr Alaini, Cairo University, Cairo, Egypt

**Keywords:** Single port robotic kidney transplantation, Single port donor nephrectomy RAKT, Living donor, Perioperative outcomes, Robotic surgery

## Abstract

Single-port (SP) robotic platforms are enabling new approaches to living donor nephrectomy and kidney transplantation, but limited access to robotic systems may prevent simultaneous donor and recipient procedures. This retrospective cohort study evaluated the feasibility, safety, and efficiency of a simultaneous SP robotic workflow using a single robotic platform for both operations. 45 living kidney donor/recipient operations were performed between October 2019 and September 2025. 15 pairs underwent a simultaneous SP robotic approach in which both donor nephrectomy and recipient transplant were completed using the same SP robotic platform, while 30 cases underwent laparoscopic donor nephrectomy followed by SP robotic kidney transplantation. Outcomes included operative time, graft ischemia times, perioperative complications, pain scores, length of stay, and graft function. Baseline donor characteristics were similar between groups. SP robotic donor nephrectomy had longer operative time than laparoscopic nephrectomy (median 4:10 vs. 3:07 h, *p* = 0.001), while warm ischemia time, blood loss, length of stay, and renal function were comparable. Donors in the SP group reported lower postoperative day 1 pain scores (*p* = 0.03). In recipients, the simultaneous SP robotic approach resulted in a shorter operative time (3:54 vs. 4:41 h, *p* = 0.015) and anastomosis time (44 vs. 52.5 min, *p* = 0.007), with no differences in cold ischemia time, complications, or graft function. Overall, this study presents a novel, resource-efficient surgical model that enables dual robotic procedures using a single platform without compromising outcomes.

**Supplementary Information:**

The online version contains supplementary material available at 10.1007/s11701-026-03643-8.

## Introduction

Kidney transplantation continues to be a cornerstone therapy for patients with end-stage renal disease, offering superior long-term outcomes compared to maintenance dialysis [1–3]. Alongside advancements in immunosuppression and recipient care, surgical techniques in both donor nephrectomy and recipient transplantation have undergone significant refinement [4–9].

Living donor nephrectomy occupies a unique ethical and clinical space as it involves operating on healthy individuals who receive no direct medical benefit. As such, minimizing donor morbidity through minimally invasive techniques and optimizing recovery has become a major focus in transplant surgery [10–14]. Similarly, the recipient operation has also advanced with the increasing adoption of minimally-invasive and robot-assisted approaches [15–19].

The introduction of the single-port (SP) robotic platform (Intuitive Surgical, Sunnyvale, CA, USA) represents a significant step forward, offering the potential for fewer incisions, improved cosmesis, and enhanced recovery for both donors and recipients [6, 9, 19, 20].

Ideally, both the donor and recipient should benefit from the advantages offered by robotic platforms. While having two individual robots would allow for true simultaneous surgery, this is not always feasible in most transplant centers due to limited robotic access and increased cost [21, 22]. As such, developing a safe and efficient workflow that enables both patients to benefit from a shared robotic system is both a practical innovation and a step toward optimizing care within existing resource constraints.

In this study, we present our experience with the first 15 consecutive kidney donor/recipient pairs performed using a single SP platform. We aim to evaluate the feasibility, safety, and reproducibility of simultaneous SP robotic nephrectomy and transplantation, while also highlighting the intraoperative logistics, perioperative outcomes, and technical considerations that may occur.

## Methods

### Study design and setting

This was a retrospective cohort study of living donor kidney transplants performed between October 2019 and September 2025 in which the recipient procedure was performed using the SP approach, either as part of a simultaneous workflow or as an isolated SP transplant. Although the donor and recipient robotic procedures were performed sequentially using a shared SP platform, elements of operative preparation and perioperative workflow overlapped temporally; therefore, the term ‘simultaneous’ was retained to reflect the coordinated donor-recipient workflow and maintain consistency with prior transplant literature. In the simultaneous SP cohort, donor nephrectomies were performed using the SP platform, whereas in the comparator cohort, donor nephrectomies were performed using a pure laparoscopic approach.

**Fig. 1:**
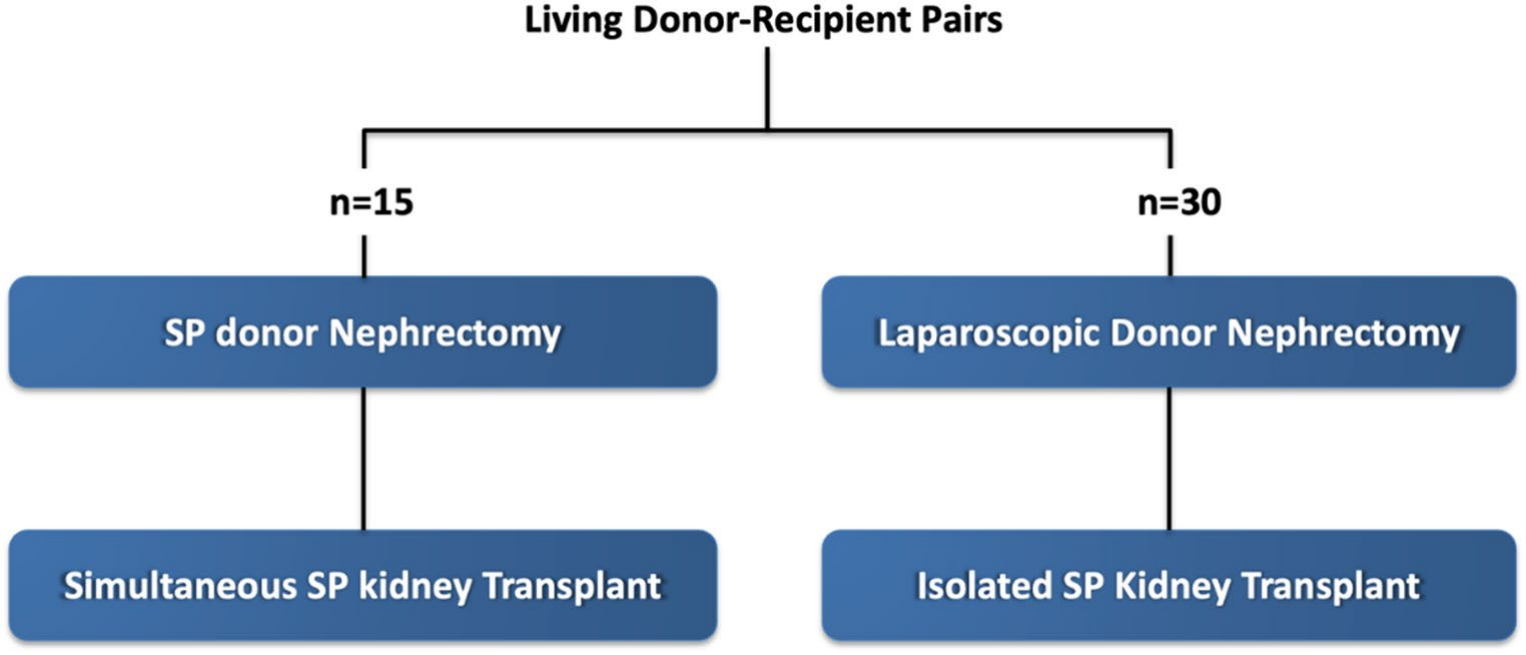
Study design and workflow of donor and recipient procedures. Living donor–recipient pairs were stratified by donor nephrectomy approach into single-port (SP; *n* = 15) and laparoscopic (*n* = 30) groups. Recipient procedures were categorized as simultaneous SP kidney transplantation or isolated SP kidney transplantation

Donor and recipient outcomes were analyzed separately. For the donor analysis, 15 SP donor nephrectomies were compared with 30 laparoscopic donor nephrectomies performed during the same period. For recipient analysis, 15 simultaneous SP kidney transplants were compared with 30 isolated SP kidney transplants (Fig. 1).

### Surgical technique

The overall workflow, including donor and recipient procedures and robotic transition, is illustrated in the video (Online Resource 1).

In simultaneous SP transplantation, both donor nephrectomy and recipient procedures are performed using the SP robotic platform. All SP donor nephrectomies were performed via transperitoneal approach while all SP transplant procedures were performed extraperitoneally. Procedures are conducted in adjacent operating rooms, with the donor surgery initiated first. In the recipient operating room, the patient is brought in approximately 30–45 min prior to kidney extraction. Anesthesia is induced, and the patient is prepped and draped. A midline or Pfannenstiel incision is performed, followed by blunt development of the extraperitoneal space and SP port placement.

**Fig. 2:**
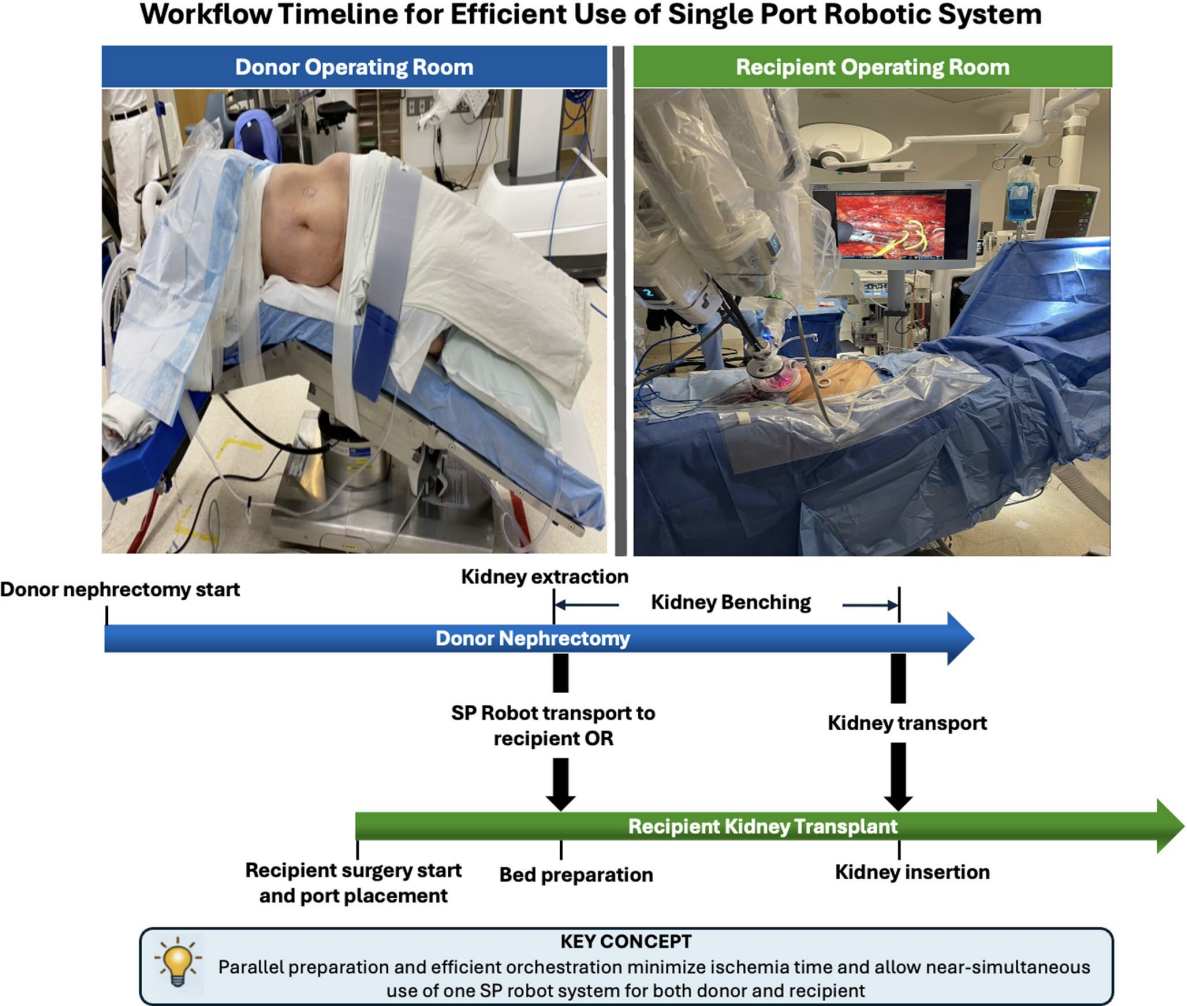
A schematic representation of the operating room layout and robotic transition process is simultaneous SP donor-recipient procedures using the same robotic platform

After kidney extraction, the graft undergoes back-table preparation per our robotic protocol (typically 20–40 min). During this interval, the donor is closed, and the robotic system is relocated and docked in the recipient’s operating room, allowing the transplant to proceed using the same SP platform. A schematic of the operating room setup and robotic transition is shown in Fig. 2.

In the isolated SP group, donor and recipient procedures were performed independently. The recipient was typically brought into the operating room approximately 1 h prior to kidney extraction. Following donor nephrectomy and back table preparation, the graft was transferred to a separate operating room where the SP robot had already been docked for vessel bed preparation and the subsequent transplant operation.

### Data collection

Demographic, clinical, and perioperative variables were retrospectively collected from the electronic medical record. These included donor and recipient age, sex, body mass index (BMI), preoperative creatinine, dialysis status, and history of prior abdominal surgery, as well as operative metrics.

Renal function in donors and graft function in recipients was assessed using serum creatinine. In donors, creatinine was recorded at discharge, 2 weeks, and 1 month postoperatively. In recipients, creatinine was recorded at discharge and 3 months post-transplant. Delayed graft function (DGF) was defined as any dialysis within the first postoperative week.

Key surgical parameters, including warm ischemia time (WIT), cold ischemia time (CIT), anastomosis time, and total operative time, were recorded. Total operative time was defined as the interval from skin incision to skin closure. Postoperative pain was assessed using the visual analog scale (VAS).

### Statistical analysis

Baseline demographic and clinical characteristics were compared between donor groups (SP donor nephrectomy vs. laparoscopic donor nephrectomy) and recipient groups (simultaneous SP vs. isolated SP kidney transplantation). Continuous variables were expressed as medians (interquartile range [IQR]) and compared using the Mann–Whitney U test. Categorical variables were compared using the chi-square or Fisher’s exact test, as appropriate.

All analyses were performed using JASP (version 0.19.3; Amsterdam, Netherlands) and R (version 4.5.2; R Foundation for Statistical Computing, Vienna, Austria). A two-sided p-value < 0.05 was considered statistically significant.

## Results

A total of 45 living donor kidney transplants were analyzed: 15 simultaneous SP donor–recipient cases and 30 cases with laparoscopic donor nephrectomy followed by isolated SP kidney transplantation.

### Donor characteristics and outcomes

Donor characteristics and outcomes are summarized in Table 1. Baseline characteristics were comparable between groups. The median donor age was 44 years (IQR 38.5–53.8) in the SP donor nephrectomy group and 49 years (IQR 38–60.5) in the laparoscopic donor nephrectomy group (*p* = 0.56). Female donors comprised 66.6% and 90% of groups, respectively (*p* = 0.06). Median BMI (29.5 vs. 27.4 kg/m², *p* = 0.20) and preoperative creatinine (0.87 vs. 0.86 mg/dL, *p* = 0.87) were similar.

**Table 1:** Donor Outcomes: Simultaneous Single-Port vs. Laparoscopic Donor Nephrectomy

Donor Data	SP Donor Nephrectomy (*n* = 15)	Laparoscopic Donor Nephrectomy (*n* = 30)	*P* Value
Age *(year*,* median*,* IQR*	44 (38.5–53.8)	49 (38-60.5)	0.56
Gender Female (*n*,* %)*	10 (66.6%)	27 (90%)	0.06
BMI *(kg/m*^*2*^, *median*,* IQR)*	29.5 (27-32.9)	27.4 (24.5–31.5)	0.20
Preoperative creatinine *(mg/dL*,* median*,* IQR)*	0.87(0.78–0.96)	0.86 (0.76–0.94)	0.87
Total Nephrectomy time (*hh: min*, median, *IQR)*	04:10 (04:02–04:19)	03:07 (02:49 − 03:29)	**0.001**
Left Kidney used *(n*,* %)*	14 (93.3%)	28 (93.3%)	1.00
**Multiple renal arteries** ***(n, %)***	0 (0%)	1 (3.33%)	0.81
Warm Ischemia *(minutes*,* median*,* IQR)*	3 (3–4)	3 (2–4)	0.70
Estimated blood loss *(mL*, *median*,* IQR)*	50 (32–50)	50 (25–50)	0.40
Postoperative Pain Score (*median*,* IQR)*			
● Day 1 ● Day 2	6 (5.5-7) 4 (3–6)	7 (6.25-8) 5 (4.5–7.25)	**0.03** 0.06
Creatinine at discharge *(mg/dL*,* median*,* IQR)*	1.26 (1.04–1.36)	1.28 (1.15–1.36)	0.46
Length of Stay *(days*,* median*,* IQR)*	2 (1–2)	2 (1.25-2)	0.56
Creatinine at 2 weeks (*mg/dL*,* median*,* IQR)*	1.16 (1.01–1.27)	1.19 (1.06–1.34)	0.10
Creatinine at 1 month (*mg/dL*,* median*,* IQR*)	1.05 (1.01–1.11)	1.01 (0.75–1.05)	0.43
Wound infection *(n*,* %*)	1 (6.66%)	0 (0%)	0.15
30-day Readmission *(n*,* %*)	1 (6.6%)	0 (0%)	0.15

Median operative time was longer in the SP donor nephrectomy group (4 h 10 min [IQR 4:02–4:19] vs. 3 h 7 min [IQR 2:49–3:29], *p* = 0.001). The proportion of left donor nephrectomy was similar (93.3% in both groups, *p* = 1.00). Median warm ischemia time was 3 min in both groups (*p* = 0.70), and estimated blood loss was 50 mL in both groups, *p* = 0.40.

Postoperative pain scores were lower in the SP group on postoperative day 1 (median 6 [IQR 5.5–7] vs. 7 [IQR 6.25–8], *p* = 0.03), with no difference on day 2 (*p* = 0.06). Length of stay was similar between groups (median 2 days in both groups, *p* = 0.56). Donor renal function was comparable at discharge, 2 weeks, and 1 month. One patient (6.6%) in the SP group developed a wound infection, while none occurred in the laparoscopic group (*p* = 0.15). Thirty-day readmission occurred in one patient (6.6%) in the SP group because of wound infection that required incision and drainage and none in the laparoscopic group (*p* = 0.15).

### Recipient characteristics and outcomes

Recipient characteristics and outcomes are summarized in Table 2. Baseline characteristics were comparable between groups. The median age was 42.8 years (IQR 32.6–58.1) in the simultaneous SP group and 50.6 years (IQR 47.6–59.9) in the isolated SP group (*p* = 0.11). Median BMI (30.3 vs. 31.4 kg/m², *p* = 0.40), rate of pre-emptive transplantation (46.6% vs. 53.3%, *p* = 0.67), and prior abdominal surgery (27% vs. 43.3%, *p* = 0.27) were similar.

**Table 2:** Recipient Outcomes: Simultaneous vs. Isolated Single Port Kidney Transplantation

Recipient Data	Simultaneous SP Transplant (*n* = 15)	Isolated SP Transplant (*n* = 30)	*P* Value
*Demographics*			
Age *(year*,* median*,* IQR)*	42.8 (32.6–58.1)	50.6 (47.6–59.9)	0.11
Gender Female *(n*,* %)*	5 (33%)	11 (36%)	0.82
BMI (*kg/m*^*2*^, *median*,* IQR)*	30.3 (25.7–33.4)	31.4 (28.5–33.9)	0.40
Pre-Emptive *(n*,* %)*	7 (46.6%)	16 (53.3%)	0.67
Cause of ESRD *(n*,* %)* - DM / HTN - ADPKD - FSGS - IgA nephropathy - Other	8 (53.3%) 0 (0%) 1 (6.7%) 2 (13.3%) 4 (26.7)	9 (30%) 6 (20%) 5 (16.6) 2 (6.67%) 8 (26.6%)	0.43
Previous abdominal surgeries *(n*,* %)*	4 (27%)	13 (43.3%)	0.27
*Operative Data*			
Total Operative time (*hh: min*, median, *IQR)*	03:54 (03:21 − 04:22)	04:41 (03:51 − 5:31)	***0.015***
Anastomosis time *(minutes*,* median*,* IQR)*	44 (39–45)	52.5 (45.5–65)	**0.007**
Cold Ischemia (*hh: min*, median, *IQR)*	01:31 (01:28 − 01:43)	01:34 (01:13 − 02:15)	0.82
Estimated blood loss *(mL*, *median*,* IQR)*	50 (50-62.5)	50 (28.5–100)	0.57
Conversion to open *(n*,* %)*	0 (0%)	1 (3.3%)	0.49
*Post Operative Outcomes*			
Length of Stay *(days*,* median*,* IQR)*	2 (2–3)	2 (2–3)	0.63
Blood Transfusion *(n*,* %)*	1 (6.6%)	4 (13.3%)	0.52
Postoperative Pain median *(median*,* IQR)* ● Day 1 ● Day 2	5 (3–6) 4 (2.5-5)	4.25 (3–6) 4 (3–5)	0.09 0.97
Creatinine at discharge *(mg/dL*,* median*,* IQR*)	1.87 (1.55–2.33)	2.54 (1.86–3.12)	0.06
DGF *(n*,* %)*	0 (0%)	2 (6.67%)	0.37
30-day Readmission *(n*,* %)*	6 (40%)	8 (26.6%)	0.28
Lymphocele *(n*,* %)*	1 (6.6%)	1 (3.3%)	0.71
Hematoma *(n*,* %)*	0 (0%)	1 (3.3%)	0.47
Creatinine at 3 months *(mg/dL*,* median*,* IQR)*	1.40 (1.21–1.47)	1.48 (1.26–1.72)	0.15

The simultaneous SP approach was associated with shorter operative time (3 h 54 min [IQR 3:21–4:22] vs. 4 h 41 min [IQR 3:51–5:31], *p* = 0.015) and shorter anastomosis time (44 [IQR 39–45] vs. 52.5 [IQR 45.5–65] minutes, *p* = 0.007). Cold ischemia time (1 h 31 min [IQR 1:28–1:43] vs. 1 h 34 min [IQR 1:13–2:15], *p* = 0.82) and estimated blood loss (50 mL in both groups, *p* = 0.57) were comparable. Conversion to open surgery occurred in one patient (3.3%) in the isolated SP group and none in the simultaneous SP group (*p* = 0.49).

Postoperative outcomes were similar. The median length of stay was 2 days in both groups (*p* = 0.63), and transfusion rates were comparable (6.6% vs. 13.3%, *p* = 0.52). Pain scores were similar between groups on postoperative day 1 (*p* = 0.09) and day 2 (*p* = 0.97).

Graft function was comparable between groups. Median creatinine at discharge was 1.87 mg/dL vs. 2.54 mg/dL (*p* = 0.06), with similar values at 3 months (1.40 vs. 1.48 mg/dL, *p* = 0.15). DGF occurred in two patients (6.7%) in the isolated SP group and none in the simultaneous SP group (*p* = 0.37).

Thirty-day readmission occurred in 6 recipients (40%) in the simultaneous SP group and 8 patients (26.6%) in the isolated SP group (*p* = 0.28). Hematoma occurred in one patient (3.3%) in the isolated SP group, while symptomatic lymphocele occurred in one patient in each group (*p* = 0.71). The reasons for readmissions are included in the supplementary table S1.

## Discussion

Kidney transplantation has undergone significant evolution over the past decade, with advances in surgical technology contributing to safer procedures, faster recovery, and improved patient outcomes [10–12, 16–18, 23]. The adoption of robotic platforms has emerged as an evolving development in kidney transplantation. The introduction of the SP robotic system enabled an extraperitoneal approach for recipient surgery, allowing patients to remain in the supine position and thereby avoiding the hemodynamic alterations associated with pneumoperitoneum and Trendelenburg positioning. Additionally, the SP platform facilitates donor surgery through a single transumbilical incision, potentially reducing morbidity. Our institution was among the first to pioneer SP robotic kidney transplantation in 2019, establishing the feasibility of this approach in the recipient population [9]. Building on this progress, Garden et al. [6] reported their initial experience with SP robotic donor nephrectomy in 2021, demonstrating the technical feasibility and cosmetic advantages of using an SP approach for healthy donors. This momentum has prompted broader exploration into how the SP platform can be applied across transplant surgery.

Implementing the SP platform for both donor nephrectomy and kidney transplantation remains challenging, particularly due to cost and availability of robotic systems [21, 22]. Most centers have access to only one SP robot, making it difficult to coordinate both procedures in close succession. This requires intraoperative planning to prevent delays that could prolong anesthesia or cold ischemia time, both associated with worse graft outcomes [24, 25]. Successful implementation depends on close team coordination, efficient OR transitions, and precise robotic management; without an integrated workflow, sharing a single robot for two complex procedures may undermine efficiency and the benefits of minimally invasive surgery.

Especially for kidney transplant donors, who are healthy individuals undergoing elective surgery, minimizing surgical morbidity and maximizing cosmetic outcomes is important. Laparoscopic donor nephrectomy is currently considered the gold standard approach. Compared with open donor nephrectomy, it provides equivalent renal outcomes while offering improved postoperative recovery [5–7]. Prior studies have shown that minimally invasive surgery for donors has increased the donation rates among kidney transplant donors [26]. The SP approach offers distinct advantages over traditional laparoscopic donor nephrectomy, which typically requires multiple trocar incisions in addition to the extraction site. In contrast, SP procedures are performed through a single, concealed umbilical incision, enhancing cosmetic outcomes, and potentially improving donor satisfaction and recovery [27]. In our donor cohort, SP donor nephrectomy was associated with a longer operative time compared to laparoscopic approach. This can be explained by the expected learning curve associated with the adoption of a new technique. Importantly, despite the increased operative duration, warm ischemia time remained comparable between groups, suggesting that graft retrieval efficiency was not compromised. Furthermore, postoperative pain scores were significantly lower on postoperative day 1 in the SP group, indicating a potential early recovery benefit. Our findings are consistent with Palese et al. [28] who reported the largest SP donor nephrectomy series to date and demonstrated reduced postoperative opioid requirements with similar pain scores and comparable perioperative outcomes between SP and laparoscopic donor nephrectomy.

From an operational standpoint, the choreography of this simultaneous approach represents a novel application of surgical logistics. The natural delay between donor kidney extraction, closure of the donor and graft implantation—typically used for bench preparation of the graft—creates a critical 30 to 40 min window [29]. Within this period, the SP robot can be undocked from the donor room, transferred, re-draped and re-docked in the recipient room, allowing the transplant procedure to begin promptly.

Our data demonstrates that recipient operative and anastomosis times were shorter in the simultaneous SP group. This likely reflects improved operating room efficiency and a streamlined workflow enabled by the coordinated simultaneous approach. In the laparoscopic donor nephrectomy cohort, the recipient robotic system was docked and prepared in advance while donor surgery was ongoing. In the simultaneous SP group, recipient induction was deliberately timed approximately 30–45 min prior to kidney extraction and was often delayed until the robotic platform became available for transfer to the recipient OR, potentially minimizing idle operative time.

Another contributing factor may be increasing institutional experience with the SP platform. By the time the simultaneous cases were performed, the surgical team had likely surpassed the initial learning curve associated with SP kidney transplantation as the first simultaneous case was performed after 37 isolated SP transplants and one isolated donor nephrectomy. Improved familiarity with instrumentation, patient positioning, and docking may therefore have contributed to reduced operative duration.

With respect to anatomical donor factors, no cases of multiple donor renal arteries were present in the simultaneous SP group, whereas one case in the comparator group involved a donor kidney with two renal arteries. Recipient factors also differed slightly between groups, as recipients in the simultaneous SP cohort were younger (42 vs. 50 years), had a slightly lower BMI (30.3 vs. 31.4 kg/m²), and had a lower proportion of prior abdominal surgeries (27% vs. 43%) compared with the isolated SP group. Although these differences were not statistically significant, they may have slightly contributed to the shorter operative times observed in the simultaneous SP cohort.

Successful implementation of this workflow requires more than access to robotic technology; it depends on a well-trained and coordinated multidisciplinary team. Technical and logistical challenges remain important considerations, as robot relocation must be performed efficiently, typically during graft preparation, to avoid operative downtime.

Our experience highlights several important considerations and potential applications. Notably, the same simultaneous approach could be adapted to the da Vinci Xi platform, offering a novel strategy to provide both donors and recipients with access to robotic surgery using a single robotic system. This concept may be particularly valuable for transplant centers with limited access to robotic platforms, as it could broaden the integration of robotic technology into both donor and recipient procedures. Additionally, this approach has implications for cost-effectiveness. By maximizing the use of a one robot across two procedures, institutions may achieve improved resource utilization and potentially reduce the cost per case [21, 22].

Although our study was not powered to detect statistical significance across all outcome measures, the observed trends in operative efficiency and early clinical outcomes are encouraging. One open conversion occurred in the isolated SP cohort, otherwise no major intraoperative complications or conversions were noted. Donor and recipient lengths of stay and discharge creatinine levels were comparable.Two lymphoceles were observed, one each in the simultaneous and isolated SP groups. This is likely a result of our SP recipient technique, in which the graft is placed extraperitoneally. Importantly, this approach was logistically sustainable across fifteen consecutive cases, supporting its reproducibility and potential applicability in other high-volume centers.

This study is limited by its retrospective design and relatively small sample size, particularly in the simultaneous SP group, which may reduce statistical power. As a single-center experience from a high-volume robotic transplant program, the findings may not be generalizable to centers with different expertise or resources. Additionally, the simultaneous approach requires specific institutional logistics, including access to adjacent operating rooms and coordinated workflows, which may limit broader applicability. Finally, long-term outcomes and cost-effectiveness were not assessed and warrant further study.

## Conclusion

This study demonstrates that a coordinated, simultaneous SP robotic approach for living donor nephrectomy and kidney transplantation is feasible and represents a novel, resource-efficient surgical model.With appropriate planning, experienced teams, and optimized workflow, this strategy may enable efficient utilization of robotic resources without compromising perioperative or transplant outcomes. In our early experience this approach was associated with improved operative efficiency, reflected by shorter recipient operative times. It offers a practical solution for centers with limited access to multiple robotic systems and has the potential to enhance operating room efficiency. Further multicenter studies are warranted to validate these findings, assess long-term outcomes, and define the broader applicability of this model.

## Supplementary Information

Below is the link to the electronic supplementary material.

Supplementary Material 1: Demonstration of feasibility and workflow for shared Single port robot performing SP donor nephrectomy via transperitoneal approach followed by SP recipient transplant via extraperitoneal approach

Supplementary Material 2

## Data Availability

No datasets were generated or analysed during the current study.
