# Supplementary material for: Feasibility of simultaneous donor nephrectomy and kidney transplantation using a shared single-port robotic platform
Source: J Robot Surg. 2026 Jul 23;20(1):743. doi: 10.1007/s11701-026-03643-8 (PMC13391760; doi:10.1007/s11701-026-03643-8)
Supplement: Supplementary file 2 — Supplementary Material 2 [file 11701_2026_3643_MOESM2_ESM.docx]

**Supplemental table**

| **Readmission reason** | **Simultaneous SP**  **n=15** | **Isolated SP**  **n=30** | **Total** | **Clavien-Dindo Grade** |
| --- | --- | --- | --- | --- |
| Cardiac (A-fib) | 0 | 1 | 1 | ≤ II |
| Fever, Diarrhea, Vomiting | 3 | 2 | 5 | ≤ II |
| GI bleed, hematoma | 0 | 1 | 1 | **≥ III** |
| Lymphocele | 1 | 1 | 2 | **≥ III** |
| Rejection | 1 | 2 | 3 | ≤ II |
| UTI | 1 | 1 | 1 | ≤ II |
| Total | 6 | 8 | 14 |  |
